# Teaching entrepreneurship in undergraduate Nursing course: evaluation of an educational proposal

**DOI:** 10.1590/0034-7167-2021-0244

**Published:** 2023-02-06

**Authors:** Rosana Maria Barreto Colichi, Wilza Carla Spiri, Carmen Maria Casquel Monti Juliani, Silvana Andrea Molina Lima

**Affiliations:** I Universidade Estadual Paulista Júlio de Mesquita Filho. Botucatu, São Paulo, Brazil

**Keywords:** Education, Nursing, Learning, Entrepreneurship, Students, Nursing, Nursing, Educación en Enfermeira, Aprendizaje, Contrato de Riesgo, Estudiantes de Enfermería, Enfermería, Educação em Enfermagem, Aprendizagem, Contrato de Risco, Estudantes de Enfermagem, Enfermagem

## Abstract

**Objective:**

To evaluate a proposal for teaching entrepreneurship in an undergraduate Nursing course that uses active methodologies and activities based on the theory of meaningful learning.

**Methods:**

Interventional, prospective study, with a quantitative perspective, with a total of 102 participating students, carried out from July 2017 to December 2019 at a public university in the state of Sao Paulo. Statistical analysis were performed by non-parametric Chi-square or Fisher’s exact tests, with differences considered statistically significant if p < 0.05.

**Results:**

Improvements were observed in almost all items evaluated, revealing that meaningful learning became more effective with the use of active teaching methodologies. Most students need adaptation and effort to be put into these methods.

**Conclusions:**

The proposal offers pedagogical content adaptation, specifically for nursing students. New research should expand teaching-learning techniques for the development of future nurses, preparing them adequately for the job market.

## RESUMO

**Objetivo:**

Avaliar proposta de ensino de empreendedorismo em curso de graduação em Enfermagem que utiliza metodologias ativas e atividades fundamentadas na Teoria de Aprendizagem Significativa.

**Métodos:**

Estudo intervencional, prospectivo, com perspectiva quantitativa, com 102 estudantes participantes, realizado de julho de 2017 a dezembro de 2019, em universidade pública do Estado de São Paulo. Análise estatística realizada por testes não paramétricos Qui-quadrado ou Exato de Fisher, com diferenças consideradas estatisticamente significativas se p < 0,05.

**Resultados:**

Observou-se melhoria em quase todos os itens avaliados, revelando que a aprendizagem significativa tornou-se mais eficaz com utilização de metodologias ativas de ensino; a maioria dos alunos necessita de adaptação e esforço a ser empreendido nesses métodos.

**Conclusões.:**

A proposta oferece adaptação pedagógica de conteúdos específica para estudantes de Enfermagem. Novas pesquisas devem ampliar técnicas de ensino-aprendizagem para desenvolvimento dos futuros enfermeiros, preparando-os adequadamente para o mercado de trabalho.

## RESUMEN

**Objetivo:**

Evaluar una propuesta de enseñanza sobre emprendimiento en cursos de graduación de Enfermería con metodologías activas y actividades basadas en la Teoría del Aprendizaje Significative

**Métodos:**

Es un estudio de intervención, prospectivo, cuantitativo, con 102 estudiantes participantes realizado entre julio de 2017 y diciembre de 2019, en una universidad pública del Estado de São Paulo, mediante análisis estadístico con testes no paramétricos Xi-cuadrado o Exacto de Fisher, con diferencias consideradas estadísticamente significativas si p < 0,05.

**Resultados:**

Hubo una mejora en casi todos los items evaluados, demostrando que el aprendizaje significativo es más efectivo con el uso de metodologías activas; la mayoría de los estudiantes necesita adaptarse y esforzarse en estos métodos.

**Conclusiones:**

La propuesta ofrece una adaptación pedagógica específica de contenidos para estudiantes de Enfermería. Las investigaciones futuras deberían ampliar las técnicas de enseñanza-aprendizaje para el desarrollo de futuros enfermeras, preparándolos adecuadamente para el mercado laboral.

## INTRODUCTION

The study on entrepreneurship has been advancing to overcome the concept based on the creation of new businesses and exploration of new business opportunities, with the entrepreneur still being responsible for the transformations in the organizational and social environments, enabling the progress of new technologies, new management procedures, new social and educational actions^(1)^.

Considering these new attributions and the importance of the topic, the Organization for Economic Cooperation and Development (OECD) recommends the empowerment of young people as economic, social and political agents, through policies that can favor their skills and promote their entrepreneurship. For this, it is necessary to strengthen the education system, promote continuous training, in addition to inserting in-service training in teaching, in order to prepare young people for the world of work, creating skills programs that better respond to market needs^(1)^. Also, Brazilian laws and curricular guidelines already establish relevant competences where professional nurses must be able to be managers, employers or leaders in the health team^(2)^.

However, low levels of entrepreneurial tendency have been observed among future nursing professionals^(3)^, including those in the same field of knowledge - health^(4)^. This scenario has been explained by many barriers such as the hospital care model, the physician-centered culture^(5)^, legal and regulatory issues^(6)^, in addition to those related to ethical and cultural values^(5, 7)^. However, recent research shows that the biggest barrier perceived by students has been the absence of entrepreneurial education in undergraduate nursing^(3)^. In addition, the institutional environment and its own structures would stop entrepreneurial talent in less traditionally entrepreneurial subjects, such as nursing^(8)^, not fully responding to the future needs of the profession regarding the expansion of the labor market.

A study carried out in Latin America and the Caribbean revealed, through the analysis of the curricula, the heterogeneity in nursing education and the focus on health care at the hospital level^(9)^. In Brazil, the National Curriculum Guidelines for the undergraduate Nursing course address administration and management skills, including the professionals’ aptitude to be entrepreneurs^(2)^. However, even with principles linked to the public health system, the proposal shares space with training trends oriented by the market and competition^(10)^. Still, little is known about the actual inclusion of entrepreneurship in the curriculum of the courses offered.

Thus, the inclusion of entrepreneurship in undergraduate nursing course has been identified as essential in the development of future professionals, allowing reflection on creative and innovative attitudes, with autonomy and determination, for the excellence of nursing and health care^(11, 12)^.

For a long time, theories about the need to have an entrepreneurial personality were considered. Nowadays, neuroscience research claims that even in the absence of biological predisposition, it can be modified by the experiences, since the brain would be able to adapt to the environment^(13)^. Thus, the development of the entrepreneurial profile at graduation becomes possible, especially if carried out in a transversal way, with the appropriate structure and the use of pedagogical methods and projects appropriate to the students^(12, 14)^. In addition, participation in educational entrepreneurship activities, the acquired business knowledge and the institutional environment would contribute positively to the entrepreneurial intentions of students^(15)^.

A recent literature review pointed out that active learning methodologies are able to place students at the heart of the learning process, favoring critical thinking and the ability to make decisions, characteristics that are essential for nurses and entrepreneurs. Addressing the use of varied strategies such as simulation, problem-based learning and inverted class, among others, the study also points out its use in integrated curricula or specific subjects. In addition to promoting the integration of theory and practice, activities carried out in groups could contribute to the training of nurses, preparing them for teamwork, a quality inherent to any good manager^(16)^.

Despite the importance and inherent characteristics of the profession, there is a scarcity of reports about methods and contents of teaching entrepreneurship that are suitable for undergraduate nursing. Considering that the use of active learning methodologies in nursing education has been adopted worldwide^(16)^, reports and experiences of educational proposals for teaching entrepreneurship with these pedagogical adaptations can encourage the use of these approaches in undergraduate courses, justifying this study.

## OBJECTIVE

To evaluate a proposal for teaching entrepreneurship in an undergraduate Nursing course that uses active methodologies and activities based on the theory of meaningful learning.

## METHODS

### Ethical aspects

This research project was approved by the Research Ethics Committee of the Faculdade de Mediana de Botucatu, UNESP, in accordance with Resolution No. 510/2016 and 466/2012 – CNS. All study participants read and signed the Informed Consent Form, securing the anonymity of the participants.

### Design, period and study setting

This is an interventional, prospective study, with the adoption of a quantitative perspective, with an empirical analytical character, guided by the STROBE checklist, carried out in the undergraduate Nursing course of a public university in the state of Sao Paulo, from July 2017 to December 2019.

### Population; inclusion and exclusion criteria

Second- and third-year students made up the study population, comprising men and women who voluntarily agreed to participate in the research.

### Study protocol

The developed stages of the study began with the identification of the teaching-learning process of entrepreneurship and the selection of differentiated pedagogical strategies, taking into account the literature review, the research developed on the subject, the recognition of the field, the organizational structures and physics of the university, as well as the curricular documents such as the Pedagogical Project of the Course and the curriculum of the subjects of Introduction to Administration in Nursing and Administration in Nursing.

The theoretical framework used was the Theory of Meaningful Learning, proposed by the psychologist David Ausubel. This theory considers that, for the teaching process, it is necessary to make some sense to the learner, and in this process, the information must interact and be based on relevant preexisting concepts in the student’s structure, that is, in the set of knowledge that the student has^(17)^.

For the evaluation, the data collection instrument was developed by the researchers, and consists of three parts: a) sociodemographic data; b) evaluation of the entrepreneurship content taught to the student, enabling the expression of criticism and suggestions for the improvement of teaching, in addition to the self-assessment of students’ participation in classes; c) worksheet to be filled in with the maximum number of possibilities for areas of activity in relation to the nurse’s job market.

The students were invited at the end of the last class of the respective semester, explaining their voluntary participation in the study. After filling out the forms, which took about 20 minutes, the forms were collected.

### Data analysis

The collected data were tabulated and organized in a database, in Microsoft® Excel program, to calculate the simple and relative frequency distribution of the variables. To assess the impact of changes in the teaching method, the second and third years of the 2019 course were compared with the second and third years of 2018 using the non-parametric Chi-square or Fisher’s Exact tests. Differences were considered statistically significant if p < 0.05. Analyzes were made with SPSS 21 software.

## RESULTS

In the studied institution, the entrepreneurship content has been inserted in the nursing graduation since 2017, in the subjects of Introduction to Nursing Administration and Nursing Administration, respectively in the second and third years. The classes, initially divided into two modules of four hours each, aimed to introduce basic notions of entrepreneurship, provide reflection on the profile of the entrepreneurial nurse and awaken the future nurse to diversified job markets.

With about 30 students enrolled in each class, the classes were held in person in rooms with university chairs, multimedia projector, whiteboard and free wi-fi internet access. Practical activities such as interviews were carried out remotely by the students and presented in the format of discussion groups.

The proposal was developed gradually, observing the best alternatives and adapting the content to this population (nursing students, mainly women), in a continuous and permanent process. This fact leads us to the prerogative that it is still under construction and must be frequently evaluated. Lectures were replaced by reflective dialogues and discussions, in addition to activities based on active methods. Here are some of the activities carried out, whose results were more significant:

1. Philosophical reflections on the social roles of employment and company, starting from images related to human needs, the female subject and others related to the desire to care and satisfaction at work, encouraging the participation of students in the search for correlations between the desire to be able to establish a social objective beyond the employment contract. Thus, providing a broad environment for discussion among the participants, avoiding the censorship of thoughts different from those normally related to care, social appeal and volunteering, the nursing vision can be expanded as a profession, overcoming conflicts, where it is possible to obtain support (salary and profit) as well as personal satisfaction (pleasure). In this activity, the barriers encountered by the groups were considered, which for the most part, are resistant to talking about profits and financial resources. According to some authors, this is due to the presence of ethical conflicts, often reinforced in educational institutions, since nursing services would be more related to volunteering than profit, a distorted view of nursing as a profession^(5)^.
2. Reflections on the current job market, based on new paradigms about public management and outsourcing of public services, in addition to increased competition for public employment. In addition to the new social dynamics, globalization and technologies as possibilities to be explored by future nurses, notions of public administration, fiscal responsibility, outsourcing and management contracts through Social Health Organizations (SHO) are discussed and explained, which change the labor relations of nurses. Clarification of students’ doubts about the future of their careers is provided, given the pretensions found with them who aim for the public tender and the hospital service as the only possible future at the end of their training^(3)^, in addition to the frequent reinforcement in institutions by legal requirement^(18)^; work in public institutions would also be related to the desire for job security, due to the financial instability of the countries^(19)^.
3. Application of students’ self-assessment of predominance of the use of abilities related to the right and left hemispheres of the brain, using the test proposed by Marquis & Huston^(20)^. Discussions are held at the end of the test, with the active participation of students, aimed at a better understanding of the differences between personal profiles and possible implications for teamwork, which can be useful in organizational processes, such as communication, decision making and problem solving^(20)^.
4. Dialogued class on concepts of entrepreneurship in nursing, still based on the characteristics of nurses, which are the same as entrepreneurs, that is, it goes beyond the notion of responsible for opening new businesses, but goes through intrapreneurship, social entrepreneurship or even academic entrepreneurship, with data from current research being presented^(3, 4, 12, 19)^.
5. The discussion about the entrepreneurial profile is based on the presentation of part of the movie “Central do Brasil”, by Walter Salles^(21)^, in which the main characters are led to think about strategies for survival and create a service to the local community. In this activity, students are invited to interact by suggesting other films and commenting on characteristics of the entrepreneurial profile, the search for solutions and the use of opportunities, reinforcing their observation skills and prior knowledge.
6. Consultation of data available on the internet of activity/company/business managed by nurses, with the purpose of reflecting on the variety of areas of activity. Conducted by the students during the class, the findings bring richness to the discussion. The activity seeks to broaden the view of the job market of future nurses, as well as to overcome barriers such as the centered medical model and hospital care that direct students to the hospital service as the only possible future at the end of their training^(5, 19)^.
7. Based on research carried out among students that revealed the need to teach notions of entrepreneurship^(3)^, and confirmed by the students’ lack of knowledge of basic notions on the subjects, the dialogue class on business formation addresses basic knowledge such as types of companies and Brazilian registration systems, as well as the planning and coordination necessary for business success, reducing risks inherent to entrepreneurship. The information discussed justifies activity 8.
8. The interview with a business entrepreneur nurse, chosen by the student, expands the concepts covered in class, enables the understanding of the importance of a business plan and contact with other market possibilities of work beyond the hospital environment and basic health units, also directing their questions to understand their experiences, whether positive or negative. It was elaborated from the request of the students during a discussion about the barriers to entrepreneurship in nursing, and the lack of contact between them with entrepreneurial professionals during their training was reported^(19)^.
9. Classes are interspersed with group dynamics and games, as well as individual challenges, always focused on the development of creativity. In addition to the playful nature, these activities were inserted from studies carried out among nursing students that revealed a low tendency to creativity, an item that makes up the Global Entrepreneurial Trend, corroborated by national and international research^(12)^.
10. The evaluation of entrepreneurship classes is carried out by the student, allowing the expression of criticism and suggestions for the improvement of teaching, in addition to the self-assessment of their participation in the classes. The main objective of the activity was to assess whether the methodologies were appropriate for the proposed educational objectives and to observe whether students began to include the possibility of entrepreneurship as a future job market. A total of 102 students participated in the evaluation, most of them were women (90%), and aged up to 21 years old (69%).

**Figure 1:**
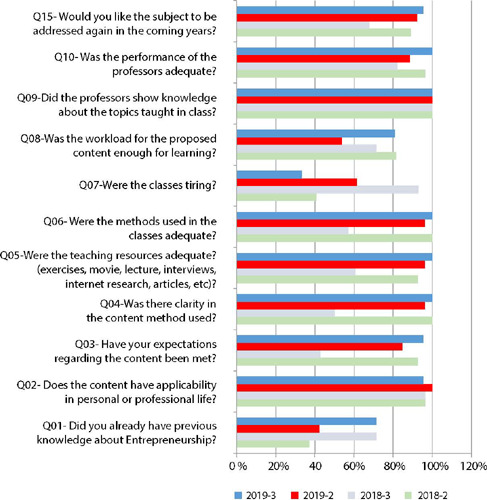
Distribution of variables evaluated by students in relation to classes, Botucatu, São Paulo, Brazil, 2018/2019

From Figure 1, it is possible to see that most of the second-year students had no prior knowledge on the subject, considered the content applicable in their professional life, had their expectations met and would like the subject to be addressed again during the course. Items related to professors, methods, teaching resources and clarity of approach were generally well evaluated by students. However, there was an indicator with a lower degree of satisfaction in relation to the workload and fatigue in classes. Reduced indicators were observed in the third year of 2018 in almost all items and that these values increased considerably in the following year (2019-3 year).

In the students’ performance, we observed problems reading bibliography and absence from classes. In addition, some of the students recognize the lack of interest in the topic and their non-active participation in activities and discussions in the classroom. However, after changes in the methods, a sharp increase in these two indicators was found in the 2019-3 class, compared to the 2018-3 class.

Regarding the possibilities of the job market mentioned by the students, it was found that there is a consensus in relation to home care by the use of the term homecare, in all classes, as shown in Figure 3. New fields emerged in 2019, with citation of practice activities related to floral therapies and aromatherapy, acupuncture, development of products and technological systems, doulas, aesthetics, dressings, offering courses or even caring for older adults, with the opening of long-stay institutions for them (ILPI’s).

**Figure 2:**
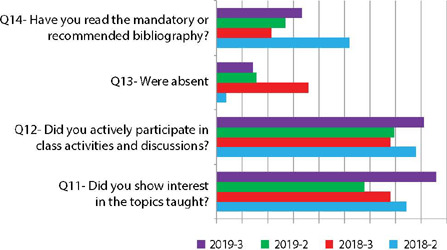
Distribution of students’ self-assessment variables, Botucatu, São Paulo, Brazil, 2018/2019

**Figure 3:**
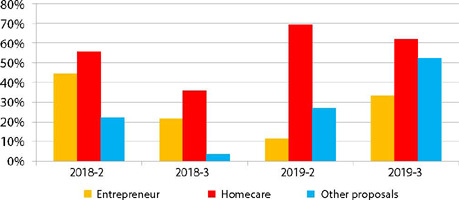
Distribution of words related to entrepreneurship cited by students, Botucatu, São Paulo, Brazil, 2018/2019

The analysis shown in Table 1 reveals that, with the sample maintaining the same demographic characteristics, there were statistically significant positive differences with the adoption of new methods and improvement in almost all items evaluated.

**Table 1:** Comparison of the impact of methods by student assessment, Botucatu, São Paulo, Brazil, 2018/2019 (N=102)

	Second year							Third year						
	2018 - 2^nd^ year			2019 - 2^nd^ year			*p* value	2018 - 3^rd^ year			2019 - 3^rd^ year			*p* value
	Total	n	%	Total	n	%		Total	n	%	Total	n	%	
Demographic aspects														
Men	27	3	11.1	26	3	11.5	1,000	28	2	7.1	21	2	9.5	1,000
Age														
16 to 19 years old		7	25.9		10	38.5			0	0.0		0	0.0	
20 to 25 years old		17	63.0		16	61.5	0.170		27	96.4		19	90.5	0.569
> 25 years old		3	11.1		0	0.0			1	3.6		2	9.5	
Evaluated questions - classes														
Q1	27	10	37.0	26	11	42.3	0.695	28	20	71.4	21	15	71.4	1.000
Q2	27	26	96.3	26	26	100.0	1.000	28	27	96.4	21	20	95.2	1.000
Q3	27	25	92.6	25	22	88.0	0.662	28	12	42.9	21	20	95.2	< 0.001
Q4	27	27	100.0	26	25	96.2	0.491	28	15	53.6	21	21	100.0	< 0.001
Q5	27	25	92.6	25	24	96.0	1.000	28	17	60.7	21	21	100.0	0.001
Q6	27	27	100.0	26	25	96.2	0.491	28	16	57.1	21	21	100.0	< 0.001
Q7	27	11	40.7	26	16	61.5	0.173	28	26	92.9	20	7	35.0	< 0.001
Q8	27	22	81.5	26	14	53.8	0.042	28	20	71.4	21	17	81.0	0.517
Q9	27	27	100.0	26	26	100.0	1.000	28	28	100.0	21	21	100.0	1.000
Q10	27	26	96.3	26	23	88.5	0.351	27	23	85.2	21	21	100.0	0.121
Q11	27	20	74.1	25	15	60.0	0.280	28	19	67.9	21	18	85.7	0.192
Q12	27	21	77.8	26	18	69.2	0.544	28	19	67.9	21	17	81.0	0.348
Q13	27	1	3.7	26	4	15.4	0.192	28	10	35.7	21	3	14.3	0.114
Q14	27	14	51.9	26	7	26.9	0.064	28	6	21.4	21	7	33.3	0.350
Q15	27	24	88.9	26	24	92.3	1.000	27	19	70.4	21	20	95.2	0.058
IND_ENT	27	12	44.4	26	3	11.5	0.014	28	6	21.4	21	10	47.6	0.070
IND_HOME	27	15	55.6	26	18	69.2	0.398	28	10	35.7	21	13	61.9	0.088
IND_OLDER ADULTS	27	8	29.6	26	5	19.2	0.379	28	15	53.6	21	11	52.4	0.934
IND_OTHERS	27	6	22.2	26	7	26.9	0.691	27	1	3.7	21	11	52.4	< 0.001

Considering the assessments, suggestions and criticisms, in addition to the incessant search for educational proposals that adequately corresponded to the students’ needs, at the end of the studied period, it was possible to outline the strategies and contents addressed, as shown in Chart 1.

**Chart 1:** Scheme of the content of the classes at the end of the period studied, Botucatu, São Paulo, Brazil, 2018/2019

Module 1 (introduction - 12h/y)	Module 2 (deepening - 12h/y)
Presentation and philosophical discussion about social roles (employment contract + social contract) Entrepreneurship concepts Contextualization of the nursing profession in the new models of state management Presentation of research on low TEG in nursing students in the region; Comparison between health professions, ILPI’s Interleaved group dynamics activities (openness to new challenges, creativity, personal differences, etc.) Presentation of the movie “Central do Brasil” and discussion; Consultation on the internet of activity/company/business managed by a nurse and presentation by the groups Entrepreneur interview and group presentation - entrepreneur profile Article reading (integrative review on entrepreneurship in nursing) and group discussion Classroom assessment and self-assessment	Review of entrepreneurship concepts Burnout syndrome and nursing career possibilities Types of companies, company registrations Introduction to the business plan: environment analysis; business areas; financial planning, structure, etc. Longevity economics (themes on population aging and ILPI’s) Interleaved group dynamic activities (openness to new challenges, creativity, personal differences, concentration, etc). Search for alternatives to barriers to entrepreneurship (based on referenced articles) – group discussion and presentation Entrepreneurial nurse interview and presentation to the room – group presentation. Classroom assessment and self-assessment

## DISCUSSION

Our experience presented an educational proposal for the teaching of entrepreneurship, offering pedagogical adaptation of contents and specific activities to nursing students, a population that often faces structural, governmental and especially cultural and ethical barriers to undertake. The evaluation showed that meaningful learning becomes more effective with the use of active teaching methodologies. However, most students need the adaptation and effort that must be put into these methods. In addition, the entire teaching-learning process must be accompanied by permanent evaluation, being a process of constant improvement.

The difficulty in raising awareness that administration and entrepreneurship are as fundamental for professional practice as clinical procedures reveals the importance of approaching the topic in nursing undergraduate courses, as soon as possible^(19)^. In addition, for nursing students, there is a need to search for their own methods, different from traditional teaching^(17)^. Thus, greater adherence to the subject was obtained when preceded by a philosophical discussion, seeking to minimize the ethical conflicts involved in a care profession. There is a need to make clear that when entrepreneurship is not necessary to move away from care, but to reinforce the exercise of these activities as a profession and have the right to receive an adequate payment for them. The proposed activity goes beyond the dualities by reflecting the dimensions of the work category, discussed by Karl Marx and the advances proposed by Hannah Arendt, for a better understanding of it as subsistence, as well as work as a result of actions that aim to overcome the earthly existence of the individual^(22)^.

Even though some of the students recognize the lack of interest in the topic, there was a marked increase in active participation in activities and classroom discussions when updated data on entrepreneurship in nursing, adapted activities and contact with entrepreneurial nursing professionals were inserted, bringing meaning to learning^(17)^.

A clear example was observed in the third year of 2018 during the business plan activity, which despite being considered common in courses such as administration, it did not prove to be effective for this audience (nursing students), due to its complexity and the detachment still present from the theme in the students’ lives. However, when replacing the activity in the following year with direct contact with entrepreneurial nurses, through interviews and visits to the respective companies, including the business plans employed, learning took place more effectively, in addition to being pleasurable, as reported by the students.

Thus, the interview, as an active method of learning, brought meaning to the proposed experiences. To choose the interviewees, the students take into account social relationships, that is, indication or people of merit recognized by them, admiration for areas chosen by the entrepreneur or even levels of kinship to contact the professionals to be interviewed. In addition, when sharing the knowledge resulting from interviews with nurses from areas that are little recognized in the nursing course, an increase in new possibilities for the individual job market, not mentioned above, is noticed.

The application of active methodologies was sought, based on the construction of collective knowledge, in which students are agents of their own learning, through research, reflection on daily practices, reading articles, interviews, group work, among others. The students become responsible for their own development, in addition to acting in the learning of the others, having to explore new approaches and contents, or even with new looks at everyday situations, aiming to understand, experience and achieve new meanings for their full development^(17)^. The teacher starts to play the role of facilitator, allowing and providing the students’ own development, leading them, contextualizing and interacting in the educational process^(17)^.

The variety of areas of activity, verified by internet research, interviews and other activities brought richness to the discussions, since entrepreneurial attitudes are not restricted to opening a business, allowing students to recognize entrepreneurship in its broadest form and to reflect on the possibility of applying it in everyday life. In addition, the need for managerial knowledge that enables business development reinforced the themes inherent to nursing administration content.

However, many students are still used to traditional methods, centered on the teacher, representing a certain resistance in carrying out more active activities and generating some strain on the teacher in an attempt to stimulate them to self-development and responsibility for their own learning^(17)^. Our results corroborate with another research among nursing students, who express difficulties in adapting to schedules that require proactive positions for their effective learning^(23)^. On the other hand, based on responses related to “tiring classes”, the insertion of playful moments between activities was provided, minimizing the effects of these situations.

Although they demand self-management skills and entrepreneurial behavior, home care activities (homecare), related to private practices, are not understood by students as businesses, denoting possible differences in the meaning of “undertaking” among this population, corroborating the notes of the GEM^(24)^ and that should be better explored in class.

Elaborated according to its diagnostic function, the evaluation should be a dialectical instrument of progress, recognition of the paths taken and the identification of the paths to be pursued^(25)^. Also recommended by the OECD, evaluating professional training and entrepreneurship programs systematically and rigorously becomes important to identify what works and what should be improved^(1)^. In addition, it is necessary to advance in the adoption of assessment by competences, focused on behavior and results, capable of developing critical thinking, addressing capacities and also constituting emancipatory assessments^(26)^.

Considering the above, classes should seek expanded concepts of discussion that involve, in addition to business entrepreneurship, intrapreneurship, social entrepreneurship and academic entrepreneurship. We suggest increasing the workload or even including a specific discipline on entrepreneurship, as well as supervised internships in private institutions, preferably constituted or managed by entrepreneurial nurses. Content on calculation methods for pricing services, analysis of new markets and costs could also be included in the programs of disciplines related to administration.

In addition, encouraging research on entrepreneurship in nursing, with the adoption of lines of research in the area, both in scientific initiation and in postgraduate studies, can contribute to deepening the theme, which is current and innovative, given the scarcity of literature and studies, mainly in Brazil and Latin America. Academic events can serve as environments for the dissemination of research carried out and for new possibilities for the nursing career.

### Limitations of the study

We point out as limitations of the study the data collection in a single educational institution, the impossibility of adopting control groups and the lack of studies related to the teaching of entrepreneurship for nursing students, making comparison with other studies difficult. Despite the limitations imposed, the study incorporates methods and new perspectives, approaching possible proposals for teaching entrepreneurship in Nursing, in the search for solutions to overcome barriers observed in the area.

### Contributions to the area of Nursing, Health or Public policy

In addition to expanding the understanding of the subject, this study can be a basis for pedagogical projects, as it addresses the teaching of entrepreneurship with pedagogical adaptation of specific contents and methods for nursing students, duly evaluated.

## CONCLUSION

The new scenarios in the labor market, with changes in public health management, the current social dynamics, as well as the most recent labor relations of nurses, have imposed the need to include topics such as entrepreneurship to better prepare future professionals during graduation, expanding their career possibilities.

So, the teaching proposal presented offers pedagogical adaptation of contents, contributing with specific methods for nursing students, a population that faces structural, governmental and cultural barriers, in addition to the ethical issues involved.

The proposal is based on meaningful learning and becomes more effective with the use of active teaching methodologies than passive learning methods, including using technologies such as cell phones and applications. However, most students need adaptation and effort that must be put into these methods.
